# Preparation, Characterization and Biological Activities of an Oil-in-Water Nanoemulsion from Fish By-Products and Lemon Oil by Ultrasonication Method

**DOI:** 10.3390/molecules27196725

**Published:** 2022-10-09

**Authors:** Nor Azrini Nadiha Azmi, Amal A. M. Elgharbawy, Hamzah Mohd Salleh, Muhammad Moniruzzaman

**Affiliations:** 1 International Institute of Halal Research and Training (INHART), International Islamic University Malaysia, Gombak 53100, Malaysia; 2 Bioenvironmental Engineering Research Centre (BERC), Department of Biotechnology Engineering, Faculty of Engineering, International Islamic University Malaysia (IIUM), Kuala Lumpur 53100, Malaysia; 3 Chemical Engineering Department, Universiti Teknologi PETRONAS, Seri Iskandar 32610, Malaysia; 4 Center for Research in Ionic Liquids, Universiti Teknologi PETRONAS, Seri Iskandar 32610, Malaysia

**Keywords:** nanoemulsion, nano-materials, ultrasonication, fish oil, lemon oil, antioxidant, antibacterial, anti-inflammatory, cell cytotoxicity

## Abstract

Fish by-product oil and lemon oil have potential applications as active ingredients in many industries, including cosmetics, pharmaceuticals and food. However, the physicochemical properties, especially the poor stability, compromised the usage. Generally, nanoemulsions were used as an approach to stabilize the oils. This study employed an ultrasonication method to form oil-in-water nanoemulsion of lemon and fish by-product oils (NE-FLO). The formulation is produced at a fixed amount of 2 wt% fish by-product oil, 8 wt% lemon oil, 10 wt% surfactant, 27.7 wt% co-surfactants and 42 min of ultrasonication time. The size, polydispersity index (PDI) and zeta potential obtained were 44.40 nm, 0.077, and −5.02 mV, respectively. The biological properties, including antioxidant, antibacterial, cell cytotoxicity, and anti-inflammatory, showed outstanding performance. The antioxidant activity is comparable without any significant difference with ascorbic acid as standard and is superior to pure lemon oil. NE-FLO successfully inhibits seven Gram-positive and seven Gram-negative bacterial strains. NE-FLO’s anti-inflammatory activity is 99.72%, comparable to nordihydroguaiaretic acid (NDGA) as the standard. At a high concentration of 10,000 µg·mL^−1^, NE-FLO is non-toxic to normal skin cells. These findings demonstrate that the NE-FLO produced in this study has significant potential for usage in various industries.

## 1. Introduction

The fish industry produces large amounts of fish by-products such as skin, heads, frames, fins, viscera, and trimmings. While always being discarded, the fish by-products have a high content of valuable nutritional compounds, including essential fatty acids. Some of these fatty acids, such as long-chain n-3 polyunsaturated fatty acids (PUFAs), are beneficial to human health [1,2] and could be a potential source of oil for various industries. Fish oil has been widely reported as a developing supplement to improve the severity of skin disorders such as dermatitis, photoaging, allergy, skin cancer, melanogenesis, and cutaneous wounds [3]. Omega-3 polyunsaturated fatty acids (PUFAs), docosahexaenoic acid (DHA), and eicosapentaenoic acid (EPA) are said to be responsible for the relationship of fish oil with skin protection and homeostasis [3]. Owing to their nutrients, fish by-products have been used to produce products for topical administration. However, fish oils are usually associated with problems such as being inherently unstable and highly susceptible to oxidation, which imperils the oil quality [4].

*Citrus limonum*, often known as lemon oil, is a highly volatile essential oil that can be applied therapeutically due to its anti-oxidative, anti-proliferative, antibacterial, and anti-cancerous merits [5,6]. Lemon essential oil’s antioxidant properties benefit human skin as it undergoes environmental and chronological ageing [7]. Other than that, lemon oil could help to restore the skin’s natural balance and nourish damaged skin while hydrating the skin. However, the physicochemical properties of lemon oil, such as high volatility, poor stability and rapid degradability upon exposure, lamentably, impede its potency [6]. Thus, to overcome the issue of oil degradation, one of the methods to stabilize the oil is through nanoemulsions formulation. Studies have shown that fish oil characteristics could be improved through this system [8,9].

Nanoemulsion (NE) is a colloidal particulate system in the submicron size range that consists of oil, water, and emulsifiers or surfactants. NE particle size can vary substantially across different studies, with particle sizes as small as 5 nm to 200 nm [10]. These carriers are solid spheres whose surface is amorphous and lipophilic with a negative charge. Their small size results in a greater surface area providing a greater absorption enabling NE to effortlessly find its way into commercial preparations in many industries. Ample studies have been done as NE has numerous advantages over other technologies, such as ease of manufacture, adjustable particle sizes, and excellent kinetic stability [11]. Since NEs consist of oil, water, and interfacial regions, lipophilic, hydrophilic, and amphiphilic components can be easily integrated into the formulation. Hence, they are suitable to be used in various products. Because of their flexibility, a broad range of delivery formulations with various functional qualities and improved sensorial properties can be developed.

Currently, low and high-energy emulsification methods can be utilized to fabricate NEs. The low-energy method transforms a water-in-oil (W/O) emulsion into an oil-in-water emulsion (O/W NE). By changing the experimental parameters, such as the composition or the temperature, phase inversion can be achieved to produce fine oil droplets at the inversion point where there is very little interfacial tension [11]. On the other hand, high-energy procedures use specific mechanical tools, such as high-pressure homogenizers, ultrasonicator, or microfluidizers, to break the droplets into extremely small particles. The ultrasonication method is frequently employed because of its appealing features where the cavitation principles are utilized. In this method, when an asymmetric cavity collapses, strong turbulence is produced by ultrasound, which starts the emulsification process by creating droplets in the acoustic field. The bigger droplets are fragmented into smaller ones, which disperse into the continuous phase as a result [8]. In recent years, research has explored ultrasonication to produce nanoemulsions with various components for various purposes. For example, Nirmal et al. [12] focused on different essential oils to produce nanoemulsions, while Nirmala et al. [13] employed ultrasonication to study the anticancer and antibacterial activities of celery oil-based nanoemulsions.

Considering that the selected oils are composed of chemical components that are sensitive to high temperatures, light, and the presence of oxygen; and that nanoemulsion systems have improved the stability and quality of many essential oils, this research aimed to produce oil-in-water nanoemulsion of lemon and fish by-product oils by ultrasonication technique for topical application. The physicochemical properties of the obtained nanoemulsions were characterized, and their antioxidant, anti-inflammatory and antibacterial activity were also assessed. This nanoemulsion produced is suitable as a topical application for skin treatment and cosmetics. It could be used as nanocarriers to improve skin penetration and provide a controlled release of active compounds for cosmetic products. Although it is not reported in this study, this formulation is currently in development for drug delivery for the rapid metabolism of active pharmaceutical ingredients and improve their bioavailability, especially the hydrophobic drugs.

## 2. Results and Discussions

An oil-in-water nanoemulsion of lemon and fish by-product oils (NE-FLO) was successfully produced, and the biological activity was tested in this study. This section reported and discussed the results obtained.

### 2.1. Organoleptic Properties of NE-FLO

**Figure 1:**
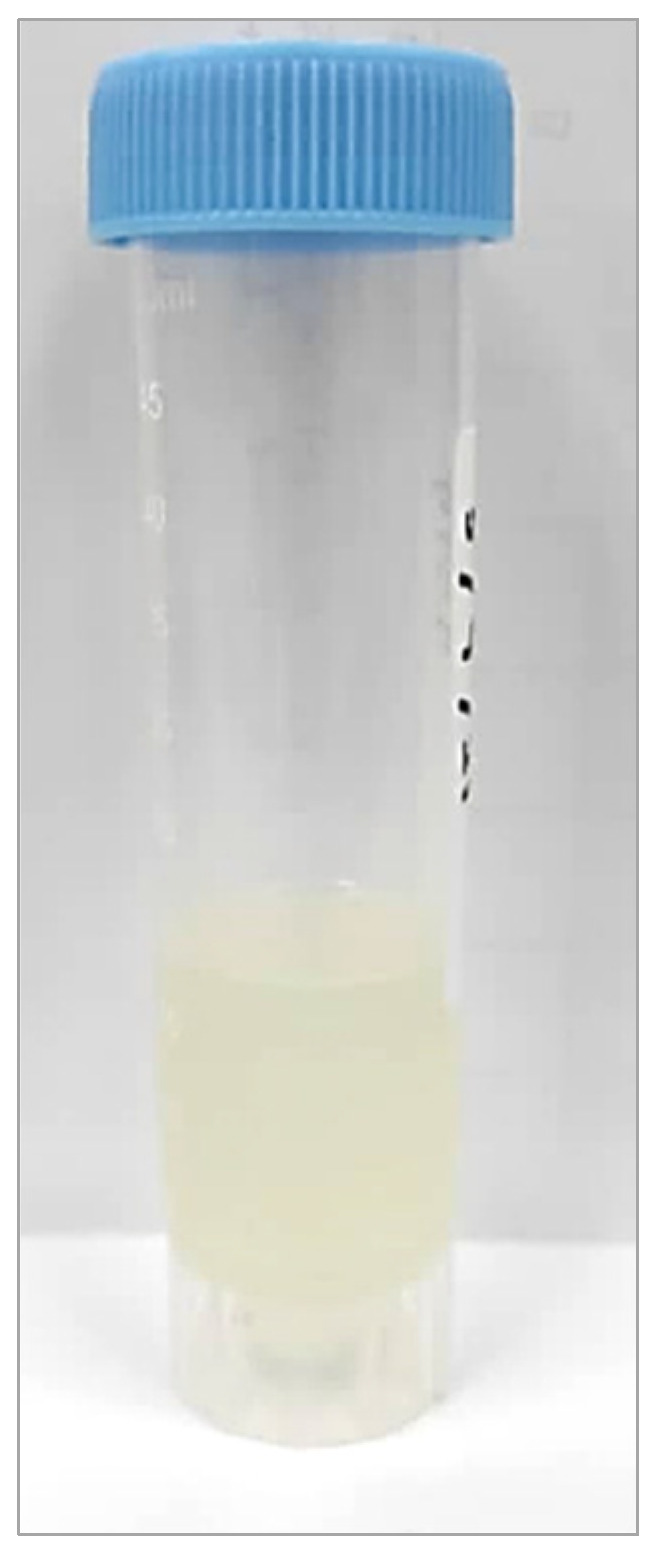
Nanoemulsion produced using ultrasonication method (NE-FLO).

Organoleptic properties are the aspects of food or other substances experienced by the senses, including taste, sight, smell, and touch. They are among the most important features to be considered in the formulation of various products as they give an aesthetic attribute to the product, which affects the customers’ choices [14]. A careful evaluation of the properties of the finished product should be conducted. This study assessed three organoleptic properties: odor, appearance, and texture. The NE-FLO exhibit a strong citrus smell owing to the incorporation of lemon oil, while its appearance exhibits translucent, yellowish color. For the texture, NE-FLO is non-sticky and has a lightweight texture which are good attributes for a product to possess. A product’s quality must be enticing to fill shelf space, capture consumer interest, and encourage repeat purchases. The performance, texture, look, and odor are among the elements that determine its quality and appeal. Figure 1 shows the NE-FLO produced by using the ultrasonication method.

### 2.2. Measurement of Particle Size, Polydispersity Index (PDI) and Zeta Potential

Photon correlation spectroscopy (PCS), also known as dynamic light scattering (DLS), was used to determine the particle size and PDI of nanoemulsions, which monitors the difference in light scattering caused by the Brownian motion of particles as a function of time. The PCS principle is based on the theory that a smaller particle will travel at a higher speed. The laser beam gets diffracted by submicron particles present in the samples. Due to particle dispersion, rapid fluctuations in laser scattering intensity arise around a mean value at a constant angle. The estimated photoelectron time correlation function generates a histogram of line width distribution that can be attributed to particle size [10,15]. Particle size distribution, mean particle diameter (Z-averages), and polydispersity index (PDI) are critical nanoemulsion quality, homogeneity, and dispersibility measures. The size for the NE-FLO recorded is 44.40 ± 0.11 nm. For most administration routes, the favorable particle size value for nanoemulsion is accepted as ≤500 nm [16]. Thus, the formulation for NE-FLO achieved the desired particle size, which is far less than 500 nm. This was also supported by a study by Rocha-Filho et al. [17], which attained an almost similar size for vegetable oil-based nanoemulsion using an ultrasonication technique. Additionally, nanoemulsions were also obtained by ultrasonication using capsaicin in the oil phase and Tween 80, glycerol in the aqueous phase where the mean droplet size is below 65 nm [18].

Reduced particle size enhanced the stability of nanoemulsions against coalescence and flocculation. When the particle size reduces, the attractive forces become weaker rapidly than the repulsive forces. Many factors are associated with the small size of nanoemulsions, such as the constitution of the formulation, production techniques, and conditions. The small particle size of NE-FLO might be due to the ultrasonication process’s energy. The primary effect of ultrasound is cavitation, which is the rapid production of vapor bubbles in a liquid at ambient temperature under decreased pressure. The resulting bubbles rapidly collapse, generating pressurized shock waves. This results in highly localized turbulence and large shear forces traversing the liquid, creating high-velocity liquid jets and breaking down the emulsion into minuscule droplets [19]. Size, on the other hand, is often not provided as a single value but rather as a representative value of a distribution that can be narrow, moderate, or broad. This means when the size of all droplets is nearly identical, the size distribution is narrow; when the size of most droplets is varied, the size distribution is broad.

The polydispersity index (PDI) in nanoemulsions is the ratio of standard deviation to mean droplet size, which reflects homogeneity in droplet size within the formulation. PDI represents the deviation from the average size. The PDI value ranges from 0 to 1, where 0 (zero) represents a monodisperse system and (1) is for a polydisperse particle dispersion [15]. A PDI lower than 0.2 is desirable as it implies that the nanoemulsion droplets are almost similar in size and free of adhesion and aggregation [19]. The PDI for NE-FLO is 0.077, indicating the homogeneity of the formulation. Ultrasonication employed by Espinosa-Andrews and Paez-Hernandez [20] produced small droplet nanoemulsions with a small polydispersity index, which is better than nanoemulsion produced by microfluidization. This study showed that ultrasonication was an efficient technique to generate nanoemulsions than microfluidization. Other studies also have shown that the ultrasonication method is good for producing nanoemulsions with a narrow polydispersity index [21,22,23]. The external power of ultrasonication used in the preparation might be the reason for the small PDI compared to the spontaneous emulsification process. Furthermore, the high concentration of surfactant and co-surfactant allows ease of dispersion, reduces aggregation, and thus, decreases PDI value.

Zeta potential is utilized to estimate dispersion stability. The value depends on many factors, such as the physicochemical characterization of the emulsion components, the presence of any electrolytes, and their adsorption ability [24]. The zeta potential’s magnitude can be used to evaluate particle stability in suspension. In theory, the molecules will repel any suspension with a highly negative or positive zeta potential value, evading aggregation and increasing the suspension’s stability. When the zeta potential obtained has an almost zero value, the surface charge is absent, which can enhance interactions between particles, facilitating aggregation or flocculation processes. Generally, for a suspension to be considered stable, a zeta potential value of greater than |30| mV should be achieved [25].

This study’s zeta potential average value for NE-FLO is −5.02 ± 0.22 mV. As a result of the addition of lemon oil and a co-surfactant into the formulation, the pH of the NE-FLO is low, which is 4.27 ± 0.01. Acidic pH can significantly decrease the zeta potential value by increasing the positive charges on the surface of the particles [26]. Conversely, at a lower pH, the electrical charge on the particles is reduced, which decreases electrostatic repulsion between them and leads to aggregation [27]. This might explain the low value of zeta potential. However, even though the zeta potential value is not in the ideal range, the charge in the nanoemulsions is sufficient to make it stable for a long time, which can be seen in the steric and electrostatic stabilization presented by colloidal dispersions [25]. This is evident in this study, where significant instability problems such as flocculation, coalescence, and sedimentation were not detected in NE-FLO at temperatures of 4 and 25 °C. Kaur et al. [6] also observed a zeta potential value lower than 30 mV, but the nanoemulsion produced had a higher stability percentage compared to the individual oil tested. Cossetin et al. [28] also encountered the same situation when their nanoemulsion zeta potential value was only 11 mV, but the formulation was remarkably stable for more than three months.

### 2.3. Morphological Analysis by Scanning Electron Microscopy

**Figure 2:**
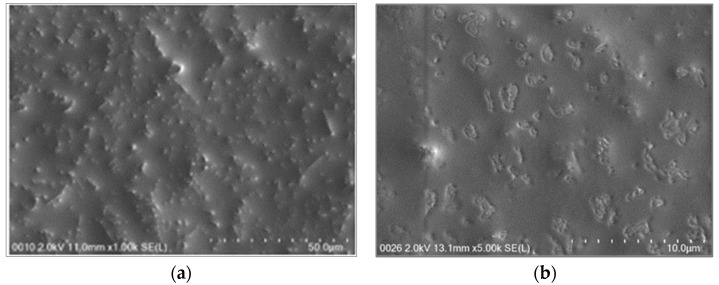
SEM images of NE-FLO in different conditions: (**a**) the SEM image taken at 1 kX magnification, (**b**) the SEM images taken at 5 kX magnification. The voltages used for both images are 2.0 kilovolts.

NE-FLO are subjected to SEM examination to study the structure and morphology at various magnifications, where the three-dimensional image of the surface morphology of the dispersed phase is created. Figure 2 shows that NE-FLO had an irregular shape with a range of 50 nm, proving the particle size recorded by the particle size analyzer. Hatziantoniou et al. [29] also demonstrated irregular shapes of nanoemulsions containing ceramide. Some studies have shown spherical shapes [30,31], which were a product of the results of this study. This might be due to the components and the way the nanoemulsions were prepared.

### 2.4. Stability Assessment

Stability studies are conducted to determine the drug substance’s stability under various environmental conditions such as temperature, humidity, and light. The impact of external factors on the quality of a drug substance or a formulated product is evaluated in a stability test, which is used to estimate shelf life, identify optimum storage conditions, and recommend labelling instructions. Stability tests of nanoemulsions can be carried out by differing the storage time or temperatures. In this study, visible observations and also physical analyses of the size and pH changes were carried out over 90 days of storage at 4 and 25 °C, respectively, to evaluate the stability of the nanoemulsions. NE-FLO still exhibits the same organoleptic qualities, such as odor, color, and texture, and there is no phase separation after 90 days. Overall, as shown in Table 1, the size of both nanoemulsions increased significantly with time (*p* ≤ 0.05). It is indeed crucial to emphasize that systems containing water, oil, and surfactant have a kinetic barrier preventing the phases from reaching kinetic equilibrium, resulting in larger droplets [28]. However, despite particle size increment, the emulsions were still in the nano range. The NE-FLO was translucent at the end of the storage period. At the end of 90 days, significant instability problems, such as flocculation, coalescence, and sedimentation, were not detected in the samples under both temperatures. It can also be observed that the room temperature conditions at 25 °C increased the particle size compared to the chilled condition. This could be due to the key mechanism of instability in these systems, the Ostwald maturation, which occurs when smaller droplets molecularly diffuse into larger droplets at higher temperatures [32].

Table 2 shows that the pH reduced significantly at room temperature on day 90. The reduction in the observed pH may again be attributed to changes in the nanoemulsion interface, which causes more contact of the oil with the aqueous phase and favours the degradation of some of its compounds and their volatilization, as was seen at elevated temperatures [28]. However, the pH remained constant throughout the 90 days of the study at 4 °C (*p* > 0.05). Maintaining the pH of a product, especially in the cosmetic market, is important. A low pH is favorable because it inhibits the growth of a wide range of bacteria in the product. Some bacteria can be dangerous and impact a product’s shelf life and stability. Products with increased pH might impact the condition of healthy skin. For cosmetic-relevant skin conditions, skin disorders and specific consumer groups, maintaining the acidic pH of the skin surface is beneficial for epidermal physiology and cutaneous microflora. Several epidermal barrier functions, like skin barrier regeneration and antimicrobial response, are related to the acidic nature of the skin surface pH. In this context, cleansing and skincare products with a pH of 4.0–5.0 may be helpful [33]. Thus, products with low pH are preferable and more helpful than a product with a higher pH.

### 2.5. Antioxidant Evaluation

The DPPH assay is one of the most utilized antioxidant assays because it is relatively simple with outstanding sensitivity. The impact of antioxidants on DPPH is based on their hydrogen-donating ability. DPPH is reduced to yield DPPH-H when an unpaired electron of DPPH is accepted by hydrogen atoms from antioxidants, causing the absorbance spectrum to change [34]. Okonogi et al. [35] interpreted inhibitory concentration (IC50) as a concentration of sample required for scavenging DPPH radical in the solution by 50%. The lesser the IC50 value, the more powerful the samples’ antioxidant activity.

From Table 3, it can be seen that there is no significant difference between the IC50 value of NE and ascorbic acid. However, the IC50 value is significantly lower than pure lemon oil.

The high antioxidant value of NE-FLO might be a contribution from the components used in the formulation, such as lemon and fish oil. The free radical scavenging activity was visible in all samples and the control, ascorbic acid. Increasing the concentration of NE-FLO from 0.039 mg·mL^−1^ to 10 mg·mL^−1^ also increased the scavenging activity.

Lemon oil, as one of the major components, might have the greatest effect on the antioxidant level of the NE-FLO. This activity may contribute to the presence of monoterpene components in lemon oil, such as limonene and terpinene, which may act as radical scavenging agents [5]. Monoterpenes and sesquiterpenes were also reported to be accountable for the neutralization of the DPPH radical [36]. However, it can be seen that NE-FLO exhibits higher antioxidants than pure lemon oil as the IC50 is lower. This observation is also supported by Liu et al. [37], in which the lemon oil nanoemulsions had better antioxidant capacities than the essential oils. It is explained that not only the primary constituents of the lemon oil should be considered, but each and every one of its constituents.

**Figure 3:**
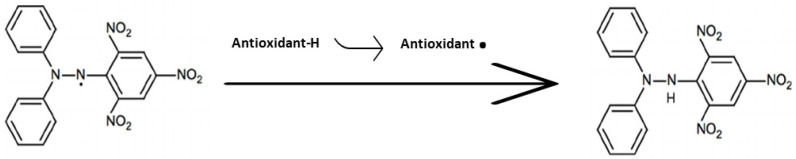
Reaction mechanism of 2,2-diphenyl-1-picrylhydrazyl (DPPH) with antioxidant.

The fish oil might also cause the higher antioxidant activity of the NE-FLO in the dispersions. The fatty acids and other compounds in fish oil, such as polyphenols, have been proven to reduce DPPH radicals [38]. In addition, the NE-FLO has a higher number of hydroxyl groups from other components than the pure lemon oil, which impacts their hydrogen-donating ability, and thus neutralizes the DPPH radical. Figure 3 shows the neutralization of the DPPH radical by the antioxidants.

Pure lemon oil was insoluble in the aqueous system, which might decrease its antioxidant activity. On the other hand, the nanoemulsions showed great water solubility, making them ideal for the efficient delivery of active components, and the huge surface area allows for excellent penetration of active chemicals [39]. Therefore, NE-FLO can more effectively scavenge radicals and have stronger reducing power, enhancing the radical scavenging capacity of the DPPH.

### 2.6. Antibacterial Evaluation

The disc diffusion approach is among the most versatile methods to measure microbes’ resistance against antibacterial agents. Generally, the filter paper disc mounted on top of the agar’s surface acts as an agent reservoir. After incubation, an inhibition area would form surrounding the filter paper disc if the samples examined were microbiologically active. The diameter of the inhibition zone appropriately defines the antibacterial ability. Nearly all of the published methods consider any inhibition zone around the filter paper disc to indicate antibiotic activity [40].

**Figure 4:**
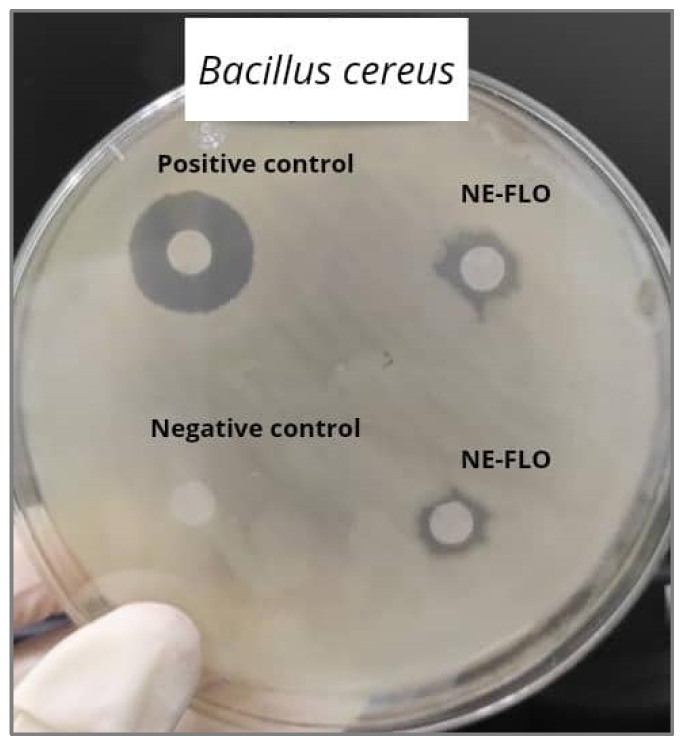
The inhibition zones for NE-FLO, the positive and negative control against *B. cereus* as, an example of the antibacterial activity of the NE-FLO.

Fourteen microbial strains were selected to evaluate the ability of NE-FLO to inhibit their growth. DMSO is used as the negative control because this universal aprotic polar solvent can dissolve NE-FLO as a test sample and is a solvent with no antibacterial properties [41]. Tetracycline is commonly employed because of its wide spectrum of antimicrobial activity, including Gram-positive and Gram-negative bacteria, spirochetes, obligate intracellular bacteria, and protozoan parasites [42]. The results in Table 4 show that the NE-FLO possesses antibacterial activity as there are inhibition zones for all plates. The biggest inhibition zone for the antibacterial activity is observed in Gram-positive bacteria, *Corynebacterium diphtheriae*. From most of the results shown, Gram-positive bacteria have a larger inhibition zone compared to Gram-negative bacteria. This might be caused by the Gram-positive bacteria morphology with a cell wall that readily absorbs the nanoemulsions, making its way easily through the inner membrane, hence interacting with intracellular sites, which is crucial for antibacterial activity [43]. Gram-negative bacteria possess high resistance to nanoemulsions’ antibacterial activity because their membrane structure is more complex [44]. Some studies reported that the lipopolysaccharides on the cell wall prohibit the nanoemulsions from penetrating and impacting the cell membrane [5,44,45]. The results presented are consistent with earlier research on citral nanoemulsions that showed significant antimicrobial activities against *Listeria monocytogenes* and *Staphylococcus aureus* [46]. Salvia-Trujillo et al. [47] also showed that the soybean oil-based nanoemulsion demonstrated bactericidal effects against *Vibrio cholerae*, *Neisseria gonorrhoeae*, *Bacillus cereus*, and *B. subtilis*. Figure 4 shows the inhibition zones for NE-FLO, the positive and negative control against *B. cereus*.

**Figure 5:**
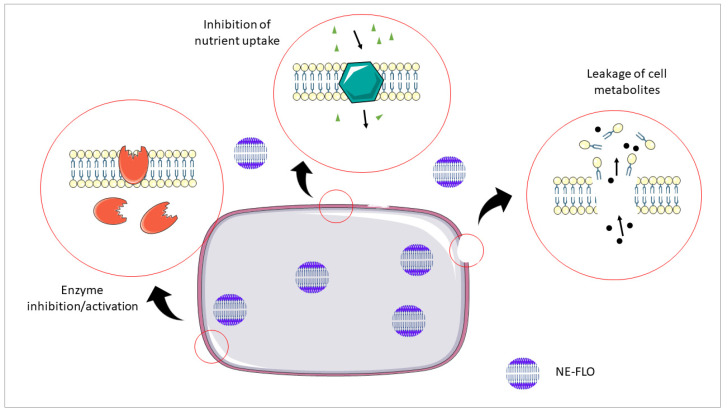
Possible mechanism of antibacterial activity of NE-FLO.

The presence of components, such as lemon oil, fish oil, and hydrophobic DES impacted the antimicrobial activity of the NE-FLO. This might be due to the fatty acids found in the fish oil, which could suppress the growth of the tested strains. NE-FLO can destroy bacteria through two means: bactericidal action and, secondly, prevent its growth (bacteriostatic action), which is reversible. In other words, in the presence of the tested samples, the bacterium cannot undergo cell division, although it remains viable [48]. Antimicrobial lipids such as fatty acids and monoglycerides have membrane-lytic capabilities due to their amphipathic qualities, which result in overlapping biophysical processes, such as membrane instability and pore formation. Membrane-destabilizing activity, in particular, induces enhanced cell permeability and lysis, resulting in bacterial cell growth inhibition (bacteriostatic action) or cell death (bactericidal action) [49]. Figure 5 shows the proposed mechanism of the antibacterial activity of NE-FLO.

### 2.7. Minimum Inhibitory Concentration (MIC) and Minimum Bactericidal Concentration (MBC) Determination

DDT only provides a qualitative assessment of the antibacterial activity; hence, the minimum inhibitory concentration (MIC) test was employed in this study to provide insight into the quantitative measurement of antibacterial activity. From Table 5, it can be observed that NE-FLO effectively inhibits the growth and killing of the bacteria. Among the bacteria tested, *Staphylococcus aureus* had the highest sensitivity since a lower concentration of NE-FLO was needed to inhibit the growth. While the highest concentration of NE-FLO (500 mg·mL^−1^) was required to inhibit the growth of *Proteus mirabilis*. This might be because of the nature of the bacteria. Gram-negative bacteria are harder to destroy because of their complex membrane structure [43]. The results from Lou et al. [39] indicated that the inhibition rate of *Staphylococcus aureus* of the nanoemulsified essential oil was significantly higher at (80.4%) than that of the pure essential oil (40.8%) at similar concentrations. This strongly suggests that incorporating the oil inside the nanoemulsion system improved the oil’s antibacterial activity.

Generally, the antibacterial activity of the nanoemulsion is said to be highly dependent on its components, the tested microbial strain, and the emulsion formulation and size. Yazgan et al. [50] also observed this, and reported that lemon essential oil-based nanoemulsion could potentially be a natural antimicrobial agent against foodborne pathogens and spoilage bacteria. It remains unknown how the antibacterial activity of nanoemulsions is exhibited. Still, the primary target is the cell membrane of the bacteria and the different basic processes inside and at the membrane. Cell lysis, destruction of nutrient absorption, production of toxic peroxidation and auto-oxidation products, and inhibition of enzyme activity are among the other mechanisms that might lead to growth inhibition or death of bacteria [44].

### 2.8. Anti-Inflammatory Evaluation

Lipoxygenase inhibition assay was used to determine the in vitro anti-inflammatory activity of the NE-FLO. NE-FLO has outstanding anti-inflammatory activity as nordihydroguaiaretic acid (NDGA), the positive control (*p* > 0.05), for the inhibition of 5-LOX activity. The results tabulated in Table 6 show that the lipoxygenase inhibition of NE-FLO is 99.72%, indicating more than 99% of the lipoxygenase enzyme are inhibited by NE-FLO. This might be due to arachidonic acid, high fatty acid content from catfish oil, and other chemical components, such as polyphenols, in the NE-FLO reported by Zadeh-Ardabili and Rad [38], who found high anti-inflammatory activity in fish and krill oil. The antioxidant activity of the NE-FLO might also be the factor for high anti-inflammatory activity [51]. 

The flavonoids and phenolic compounds responsible for the antioxidant effect might also exert an anti-inflammatory effect. Bioactive extracts and their natural constituents have been shown to exert biological characteristics by inhibiting two important signaling pathways, NF-B and mitogen-activated protein kinases (MAPKs), which are involved in the production of numerous proinflammatory mediators [52]. Randy et al. [53], who studied the anti-inflammatory activity of partially purified Bromelain-loaded nanoemulsion, also showed similar results where the bromelain-loaded nanoemulsion has the highest activity compared to commercial bromelain and the standard. The NE-FLO has thus demonstrated the potent inhibition of LOX activity due to their constituents from lemon and fish oil. Hence, it shows potential for future development into a functional cosmeceutical product for treating and preventing of inflammatory-associated diseases.

### 2.9. Cell Cytotoxicity Evaluation

The in vitro cytotoxic and anti-proliferative activity of the NE-FLO were evaluated using the MTT assay. This assay is commonly used to assess the in vitro cytotoxic effects of active drugs on cell lines or primary patient cells because the total mitochondrial activity corresponds with the percentage of viable cells in most cell populations [54]. In addition, the nanoemulsion must be biocompatible with mammalian cells for applications in cosmeceutical products. Nanoemulsions that are toxic or could release toxic by-products would destroy the skin cells and eventually worsen the conditions of the targeted area. Therefore, the toxicities of the NE-FLO were examined by indirect MTT assays using human skin fibroblasts (HSF 1184) as the model cells.

**Figure 6:**
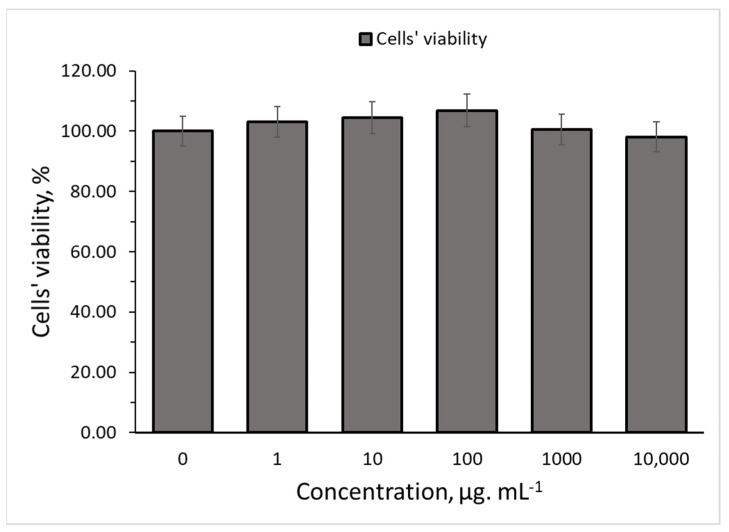
Cell viability of fibroblast cells (HSF 1184) cultured with increasing concentrations of NE-FLO using the MTT assay. Data are presented as mean ± SD (*n* = 3). Control is the cell treated with DMEM only.

Among all the treatments with different concentrations of conditioned media obtained from the NE-FLO, no significant decrease in cell viability was observed compared to control cells. The results in Figure 6 revealed that NE-FLO increased cell growth and is nontoxic toward the HSF1184 normal skin cell lines. Vater et al. [54] found that nanoemulsions containing traditional herbal plants showed higher skin cell viability in MTT tests than the control. Similar findings from Zanela da Silva Marques et al. [55] also showed that a nanoemulsion containing propranolol is nontoxic and maintains the growth of over 80% of the fibroblast cell.

Increasing the concentration of NE-FLO caused the HSF cell viability to increase as well. The highest peak was at a 100 µg·mL^−1^ concentration, which reached 106 ± 4% cell viability compared to the control. It can also be seen that even at high concentrations of 10,000 µg·mL^−1^, the NE-FLO did not display any toxic effect on the skin.

Fatty acids from fish oil might also be one of the reasons for skin cell proliferation. The fatty acids, such as palmitic acid, aided in keratinocytes and fibroblast proliferation, possibly by additionally fuelling cellular metabolism [56]. A review by Carvalho et al. [57] shows that omega-6 fatty acids have received much attention because they can modulate cell migration and proliferation, phagocytic capacity, and synthesis of inflammatory mediators, which are also found in the fish oil used in the NE-FLO. Moreover, the nano-sized emulsions could penetrate better into the cells and proliferate skin cells due to their small size [58].

## 3. Materials and Methods

### 3.1. Materials

2,2-diphenyl-1-picrylhydrazyl (DPPH) reagent and Folin–Ciocalteu reagent were obtained from Merck, Darmstadt, Germany. Sodium linoleate, soybean lipoxygenase enzyme solution and nordihydroguaiaretic acid (NDGA) for anti-inflammatory studies were purchased from Merck. Dulbecco’s Modified Eagle’s Medium (DMEM), fetal bovine serum and 3-(4,5-dimethylthiazol-2-yl)-2,5-diphenyltetrazolium bromide (MTT) were purchased from Life Technologies Corporation, Carlsbad, CA, USA. Human skin fibroblast cells (HSF 1184) were obtained from Life Technologies Bio Diagnostic, Malaysia. South African lemon oil was obtained from a local supplier (Wellness Original Ingredient, Malaysia). Catfish by-product oil was prepared in the laboratory as previously described by Djaeni and Listyadevi [59]. The anionic co-surfactants used in the study are not stated as it is under a patent application. All other solvents and chemicals used were analytical and/or pharmaceutical grade.

### 3.2. Preparation of Nanoemulsion of Fish by-Product and Lemon Oils (NE-FLO)

The method for ultrasonication is adopted from Espinosa-Andrews and Paez-Hernandez [60]. A two-step process was used to prepare the oil-in-water nanoemulsion of fish by-products and lemon oils (NE-FLO). Briefly, the continuous phase, known as the aqueous phase, was prepared by adding 10 wt% of Tween 80 and 27.7% cosurfactant to pure distilled water and stirred for 15 min. The oil phase consisted of 2 wt% fish oil and 8% lemon oil. The aqueous and the oil phases were mechanically stirred at 500 rpm until both mixtures were well combined. The oil phase was then added dropwise into the continuous aqueous phase while stirring at the same speed. A T25 digital Ultra-Turrax homogenizer was used to combine the mixture for 3 min at 20,000 rpm to form a coarse oil-in-water emulsion. The coarse emulsion was ultrasonicated at a predetermined amplitude using an ultrasonicator (LABSONIC M, Sartorius, Teltow, Germany) for 42 min to obtain the nano-sized droplet. The ultrasonication time is based on the optimization of this study. The sonication was set at 80% of the maximum output of 100 W.

**Figure 7:**
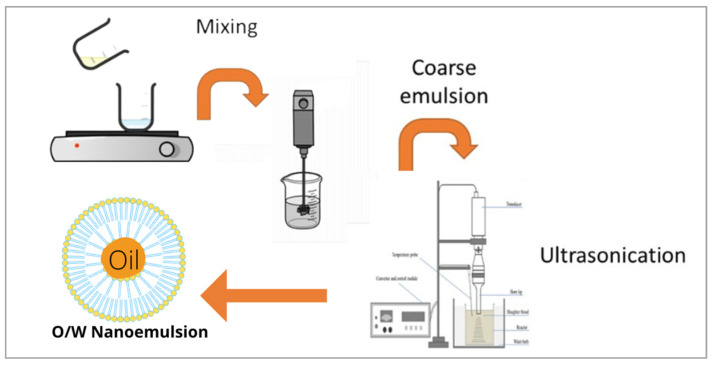
Schematic diagram of the preparation of NE-FLO using the ultrasonication method.

There is no pulse used; however, to avoid an increase in temperature, the beaker is put inside a water-filled beaker. Thus, even when the samples were sonicated for a long time, there was no increase in temperature detected. Tween 80 was used as a surfactant as it is a hydrophilic non-ionic surfactant that is well-established in the emulsification and dispersion of substances in pharmaceutical and food products. Other than that, Tween 80 was also listed as one of the generally regarded safe agents (GRAS). For the co-surfactant, it was proven that it can form hydrogen bonds with water, lowering the water−oil interfacial tensions and improving the interface’s fluidity. The percentage of the surfactant and co-surfactant used were optimized. Figure 7 shows the schematic diagram of NE-FLO preparation using the ultrasonication method.

### 3.3. Measurement of Particle Size, Polydispersity Index (PDI) and Zeta Potential

The determination of particle size, PDI and Zeta potential of NE-FLO were analyzed using a particle size analyzer (Malvern Instruments, Malvern, UK) at 25 °C. Dilution using distilled deionized water at a ratio of 1:9 of all samples was carried out to prevent back-scattering phenomena. The particles and water refractive indices employed were 1.54 and 1.33, respectively. Each measurement was repeated in triplicate.

### 3.4. Morphological Analysis by Microscopic Techniques

Scanning electron microscopy (Vega3 Tescan, Kohoutovice, Czech Republic) was used to observe the morphology of the NE-FLO following a method by Krithika and Preetha [31]. NE-FLO samples were mounted on the stabs with adhesive tape, and SEM images were captured at a 20 kV accelerating voltage with a magnification of ×100 k. To examine the NE-FLO images, multiple resolutions ranging from magnification ranging from 20× to approximately 30,000× were used accordingly.

### 3.5. Stability Assessment

The emulsion stability, or an emulsion’s ability to withstand changes in its properties over time, was studied by storing the NEs at various temperatures. Measurements were carried out at 0, 7, 14, 21, 28, and 90 days of storage. The visual instability (creaming, phase separation, or flocculation) was observed and recorded (if any). The determination of particle size of NE-FLO at day 0 and day 90 were analyzed using a particle size analyzer (Malvern Instruments, Malvern, UK) at 25 °C. The stability was also evaluated by monitoring changes in pH at different intervals of time using a pH meter (Mettler Toledo, Zurich, Switzerland).

### 3.6. Antioxidant Evaluation

The in vitro antioxidant activity of the NE-FLO was conducted using the 1,1-diphenyl-2-picrylhydrazyl (DPPH) assay. DPPH solution at an amount of 2.5 mL (60 µmol·L^−1^ in ethanol) was combined with 0.3 mL of the NE-FLO and 0.2 mL of ethanol in a 10 mL test tube with a final volume of 3.0 mL. The absorbance of the solution was measured at 517 nm after allowing the mixture to stand for 30 min at room temperature.

### 3.7. Antibacterial Evaluation

The disc diffusion test (DDT) and minimum inhibitory concentration (MIC) are the most used tests for antimicrobial properties against Gram-positive and Gram-negative bacteria. In this study, seven strains of Gram-negative bacteria and seven strains of Gram-positive bacteria were used. All the bacterial strains were first precultured by placing a loopful of cells into 10 mL of autoclaved tryptone soy broth (TSB) and incubated at 37 °C for 24 h. Then, the cells were spread evenly on the agar plates using a sterile cotton bud. A sterile filter paper (Whatman No. 1, 6 mm diameter) was immersed in NE-FLO and equilibrated before being placed on the seeded plates before incubation. The plates were then allowed for incubation (24 h, 37 °C). The diameter of the inhibition zones was measured to quantify the antimicrobial activity. The analysis was repeated in triplicate for each of the evaluated bacterial strains. The positive and negative controls used are tetracycline at 5 mg·mL^−1^ and dimethyl sulfoxide (DMSO), respectively [45].

MIC is the minimum concentration of an antibacterial agent that completely inhibits the microorganism’s growth in tubes or microdilution wells detected by the unassisted eye, and it was observed through 96-well agar plates. The antibacterial activity of the NEs was assessed with a broth micro-dilution assay, using a serial dilution in sterile 2 mL 96-well microplates [61]. Each of the rows was filled with 0.1 mL sterilized tryptone soy broth. Wells 2–11 were topped up with 0.1 mL of NE-FLO and diluted serially to form a sequence of concentrations from 500 mg·mL^−1^ to 0.488 mg·mL^−1^. The first well served as growth control. Then, 10 µL of the culture medium was placed in each well containing NE-FLO. The microplates were then incubated (37 °C, 24 h). The turbidity assessment was carried out at the time of incubation, *t*T = 0, and *t* = 24 by reading the optical density at 600 nm (UV–vis spectrophotometer, Thermo fisher, Massachusetts, USA). The analysis was done in triplicate for each assay. Minimum bactericidal concentration (MBC) is the minimum concentration of an antibacterial agent needed to kill and prevent the growth of a bacterium over a fixed and extended time, such as 18 h or 24 h, under a specific set of conditions. A loophole from each well that is equal and higher than MIC is streaked on Mueller–Hinton agar to validate the MIC value. All plates were incubated at 37 °C for 24 h. The minimum bactericidal concentration (MBC) is expressed as the concentration at which no apparent and visible growth of bacteria occurs. The test was performed in triplicates.

### 3.8. Anti-Inflammatory Evaluation

The activity of lipoxygenase inhibitors was accurately determined by slightly altering the spectrometric approach described by Azhar-Ul-Haq et al. [62]. In the protocol of the assay, 160 µL of sodium phosphate buffer (100 mM, pH 8.0), 20 µL of soybean lipoxygenase enzyme solution, and 10 µL of the NE-FLO were thoroughly mixed and incubated at 25 °C for 10 min, without shaking. The addition of 10 µL sodium linoleate (0.3 mM) as substrate solution initiated the enzyme reaction, and it was incubated once again in a spectrophotometer at 25 °C for 10 min. Linoleic acid was converted to form (9Z,11E)-(13S)-13-hydroperxyoctadeca-9,11-dienoate (13-HPODE) following the incubation period and measured by spectrophotometer at 234 nm. The initial reaction rate was measured by spectrophotometry, and the inhibitory activity of a sample was measured by the reduction of this initial rate [63]. The test compounds and the positive control were dissolved in DMSO because this universal aprotic polar solvent can dissolve NE-FLO as a test sample. All the reactions were performed in triplicates, and the absorbance was measured at 234 nm. Nordihydroguaiaretic acid (NDGA) was used as the reference standard. The percentage of inhibition was calculated using Equation (1).

% inhibition = ((OD_control_ − OD_sample_)/OD_control_) × 100 (1)

where OD_control_ is the optical density of control, and OD_sample_ is the optical density of the sample.

### 3.9. Cell Cytotoxicity Study

Cell lines used in this study, human skin fibroblast cells (HSF 1184), were cultivated in Dulbecco’s Modified Eagle’s Medium (DMEM), with 1% penicillin with streptomycin and 10% fetal bovine serum. The cells were maintained at 37 °C in a humidified atmosphere of 5% CO_2_. The reduction of MTT dye by viable cells to yield purple formazan products was used to assess the NE-FLO cytotoxicity to the cell according to the method by Azrini et al. [64]. Firstly, fibroblast cells were seeded at a density of 1 × 104 cells using a 96-well plate, followed by incubation in a culture medium until 100% confluence. Next, the varied concentrations of NE-FLO were used to treat the cells for 24 h. Different concentrations of NE-FLO were used to determine the concentration at which the NE-FLO is toxic to the cells. After 24 h, the media was withdrawn and washed with phosphate-buffered saline. The cell was incubated in the dark with 20 µL of 5 mg·mL^−1^ MTT solution for four hours at 37 °C. Next, 100 µL of DMSO was added to each well. The absorbance was measured at 570 nm using a Tecan M200 Pro Multiplate reader. The percentage of viable cells was determined with the assumption that the cell viability without treatment was 100%.

## 4. Conclusions

This study successfully produced oil-in-water nanoemulsions of lemon and fish by-product oils with the desired particle size and the polydispersity index (PDI) using the ultrasonication method. The antioxidant activity showed comparable results with ascorbic acid and was superior to lemon oil. The results also showed that NE-FLO possessed antibacterial activity against all of the bacteria strains tested in the study. NE-FLO also showed no signs of toxicity against normal skin cells. The anti-inflammatory activities of the NE-FLO were remarkably high and demonstrated no significant difference compared to the nordihydroguaiaretic acid (NDGA). The findings from the study revealed that NE-FLO could be a potential topical product for skin problems. Further research on the NE-FLO ability in cosmeceutical products should be conducted to support this study.

## Figures and Tables

**Table 1:** Stability study of NE-FLO at different storage temperatures assessed using size analysis at different storage times.

Temperature	Size Day 0 (nm)	Size Day 90 (nm)
25 °C	44.40 ± 0.11 ^a^	89.24 ± 0.025 ^b^
4 °C	44.40 ± 0.11 ^a^	125.15 ± 0.00 ^c^

Data are presented as mean ± SD (*n* = 3). Different letters in the columns indicate statistically significant values compared to day 0 (*p* < 0.05).

**Table 2:** Stability study of NE-FLO at different storage temperatures assessed using pH measurement at different storage times.

Temperature	pH Day 0	pH Day 90
25 °C	4.27 ± 0.01 ^a^	4.22 ± 0.025 ^b^
4 °C	4.27 ± 0.005 ^a^	4.27 ± 0.00 ^a^

Data are presented as mean ± SD (*n* = 3). Different letters in the columns indicate statistically significant values compared to day 0 (*p* < 0.05).

**Table 3:** The IC50 value of lemon oil, NE-FLO, and ascorbic acid used in the DPPH assay.

Samples	IC_50_ Values (mg·mL^−1^)
Lemon oil	0.424 ± 0.0013
Ascorbic acid	0.281± 0.0013
NE-FLO	0.300± 0.0565

**Table 4:** Antimicrobial activity of NE-FLO and controls against different bacterial strains by disc diffusion method.

Gram-Positive Bacteria	NE-FLO	Positive Control (Tetracycline 10 mg·mL^−1^)	Negative Control (DMSO)
*Bacillus cereus*	3 ± 0 mm	11 ± 2 mm	0 ± 0 mm
*B. subtilis*	6 ± 0 mm	18.3 ± 1.15 mm	
*Clostridium perfringens*	2 ± 0 mm	14.67± 1.15 mm	
*Corynebacterium diphtheriae*	8.67 ± 0.57 mm	19 ± 0 mm	
*Listeria monocytogene*	4.33 ± 0.57 mm	19.67± 0.57 mm	
*Staphylococcus aureus*	3 ± 0 mm	19 ± 0 mm	
*Streptococcus pneumoniae*	4.33 ± 0.57 mm	6.67 ± 1.15 mm	
**Gram-Negative Bacteria**	**NE-FLO**	**Positive Control (Tetracycline 10 mg·mL^−1^)**	**Negative Control (DMSO)**
*Escherichia coli*	4 ± 0 mm	18.67 ± 0.57 mm	0 ± 0 mm
*Proteus mirabilis*	3.33 ± 0.57 mm	19.33 ± 1.53 mm	
*Vibrio vulnificus*	4 ± 0 mm	10.33 ± 1.53 mm	
*Vibrio parahaemolyticus*	4 ± 0 mm	16.33 ± 2.5 mm	
*Salmonella enteritidis*	4 ± 0 mm	18.67 ± 0.57 mm	
*S. typhimurium*	3 ± 0 mm	18 ± 0 mm	
*Shigella sonnei*	3.33 ± 0.57 mm	5 ± 1 mm	

**Table 5:** Evaluation of Minimum Inhibitory Concentration and Minimum Bactericidal Concentration (MBC) of NE-FLO on different bacteria.

Bacteria/Test	MIC (mg·mL^−1^)	MBC (mg·mL^−1^)
*Bacillus cereus*	250	250
*B. subtilis*	250	500
*Corynebacterium diphtheriae*	250	500
*Clostridium perfringens*	125	125
*Listeria monocytogenes*	250	250
*Staphylococcus aureus*	62.5	125
*Streptococcus pneumoniae*	250	250
*Escherichia coli*	250	250
*Proteus mirabilis*	500	500
*Salmonella enteritidis*	125	250
*Shigella sonnei*	125	250
*Salmonella typhimurium*	125	250
*Vibrio parahaemolyticus*	250	250
*V. vulnificus*	250	500

**Table 6:** Anti-inflammatory activity of NE-FLO assessed using lipoxygenase inhibition assay.

Samples	NDGA	NE-FLO
Lipoxygenase inhibition (%)	99.83 ± 0.27 ^a^	99.72 ± 0.23 ^a^

The control used is the nordihydroguaiaretic acid (NDGA). Data are presented as mean ± SD (*n* = 3). The same letter in the columns indicates the values are not statistically significant compared to NDGA.

## Data Availability

Data is contained within the article.

## Sample Availability

Samples of the compounds used in this study are available from the corresponding authors upon request, subjected to availability.
